# Does the Admission Blood Pressure Associate with Short- and Long Term Outcome in Stroke Patients Treated with Thrombolysis? A Single Centre Study

**DOI:** 10.1155/2013/610353

**Published:** 2013-07-31

**Authors:** L. Bentsen, C. Ovesen, A. F. Christensen, H. Christensen

**Affiliations:** ^1^Department of Neurology, Copenhagen University Hospital, Bispebjerg Bakke 23, Bispebjerg, 2400 Copenhagen NV, Denmark; ^2^Department of Radiology, Copenhagen University Hospital, Bispebjerg Bakke 23, Bispebjerg, 2400 Copenhagen NV, Denmark

## Abstract

*Background*. The association between outcome and elevated admission blood pressure (BP) remains uncertain in acute stroke patients. The aim of the present study was to examine the association between admission blood pressure and outcome in ischemic stroke patients treated with tissue plasminogen activator (tPA). *Method*. This study included patients treated with tPA within 4.5 hours after symptom onset. Four quartiles based on the admission BP values were defined. BP development of the first 12 hours was compared to outcome parameters defined as NIHSS 24 hours after tPA and mRS after 3 months. *Results*. 265 patients were included. A trend with worse short- and long-term outcome was present in the quartiles with the lowest and highest admission BP compared to the quartile with admission values at 140–160 mm Hg systolic. BP in quartile 1 was insignificantly decreased after 12 hours while the BP in quartiles 3 and 4 remained above recommended levels. *Conclusion*. Admission BP is associated with short- and long-term outcome after stroke. Low- or high-admission BP indicates cardiac comorbidity or preexisting hypertension, where close monitoring and further examinations are requested to prevent poorer outcome.

## 1. Introduction

The relation between acutely elevated blood pressure and stroke severity and outcome in acute stroke fuels the continued debate whether the increase in blood pressure shall be seen primarily as a pathological mechanism or a physiological stress indicator.

A U-shaped relation between the level of acute elevation in blood pressure and outcome has been introduced defining a middle area with better prognosis than patients with high as well as low blood pressure [1–3]. High blood pressure is observed in the majority of patients admitted with acute stroke [4, 5]. Several factors contribute to this including stress of hospitalisation, activation of neuroendocrine hormones, and obviously hypertension [6, 7]. Thus patients with no premorbidity are considered to be able to develop a relevant stress response when afflicted with an acute vascular episode and in this group blood pressure levels decrease to normal levels within hours [8]. Patients with ischemic heart disease have compromised blood pressure reserve, and thus the lack of an adequate increase becomes a predictor for a worse outcome [9, 10]. In patient groups with preexisting hypertension a multifactorial genesis based on a combination of various risk factors and premorbid conditions results in a lack of blood pressure regulation. These patients have a further overshooting of the initial blood pressure levels which remain high throughout hospitalization and have a tendency towards a worse outcome [11]. Identification of which subgroups of stroke patients could profit from blood pressure reduction or extensive cardiac work up is being investigated in ongoing studies and can be offered alongside standard acute treatment, that is, tissue plasminogen activator (tPA) [12]. Patients selected to receive tPA constitute a homogenous group representative for those with the highest expected treatment response and thus is a core target group for additional workup like regulation of blood pressure or cardiac comorbidity. 

The aim of the present study was to examine the association between admission blood pressure levels and the subsequent development in outcome in ischemic stroke patients treated with tissue plasminogen activator (tPA).

## 2. Method

This prospective cohort study included consecutive patients treated with tPA within the first 4.5 hours after symptoms onset from April 2009 to December 2011 at Copenhagen University Hospital, Bispebjerg, which has a catchment area of 1.7 million on even dates. Inclusion criteria included reported admission values for systolic blood pressure, admission NIHSS, and prehospitalization mRS, but missing values of diastolic blood pressure, follow-up NIHSS, and mRS after 3 months were accepted due to a random pattern of undecipherable values.

On admission, patients underwent standard neurological and radiological workup including clinical scoring (i.e., NIHSS), biochemistry, and monitoring.

Radiological workup contained noncontrast computed tomography (CT) and CTA from the arcus aorta to the vertex cranium on a 64-slice MDCT scanner (Phillips).

tPA treatment decision was based on clinical and radiological findings and post-tPA follow-up included 24 hours monitoring, CT-C, and NIHSS evaluation on day 2. After discharge all patients were followed up at 3 months by telephone or in the outpatients' department. Patients' data regarding ability to be self-reliant after discharge were collected and described with modified Rankin Scale (mRS).

On admission nurses measured the blood pressure manually using standard automated OMRON sphygmomanometers with fitting cuff and the patient in a supine position and subsequent monitoring included repeated measurements 12 hours post-treatment.

### 2.1. Collected Data and Definitions

Clinical data was collected prospectively from the patient files regarding relevant blood pressure measurements, biochemistry, NIHSS, and medical history. 

Likewise the presence of relevant risk factors including hypertension, diabetes mellitus, hypercholesterolemia, prior myocardial infarction, atrial fibrillation (AFIB), smoking, and alcohol consumption was noted. 

Hypertension was defined either by registration of the diagnosis in previous medical charts, use of antihypertensive medication, or 3 blood pressure measurements ≥140/90 mm Hg taken 1 hour apart and at least 24 hour after stroke onset. 

Diabetes mellitus was defined by use of antidiabetic medication, fasting p-glucose ≥ 9 mmol/L, or HgA1c ≥ 6.5. Definition of hypercholesterolemia was use of lipid-lowering medication or p-cholesterol ≥ 5.0 mmol/L. Prior myocardial infarction or coronary bypass surgery defined ischemic heart disease and AFIB was defined by previous AFIB or ≤30 seconds or documented at ECG in 12 leads. 

Smoking was defined as current use of tobacco and former smoker was defined with a history of at least 3 pack years. Consumption of alcohol to an excessive amount was defined with ≥14/21 units of alcohol weekly, respectively, for women and men. 

We divided patients into the four quartiles of systolic and diastolic blood pressure values on admission. The blood pressure change at 12 hours was also calculated for these groups. Admission blood pressure levels as well as their degree of subsequent decline (after 12 hours) were compared to outcome parameters, which were defined as NIHHS development 24 hours after thrombolysis and 3 months outcome (mRS). A good outcome was defined as mRS ≤ 2 and a poor outcome as mRS > 2.

The presence of risk factors was compared between groups concerning significant differences.

### 2.2. Statistics

Statistics were calculated in SPSS, version 19.0. Demographic variables, risk factors, NIHSS, and mRS were compared in the four blood pressure groups with the Kruskal-Wallis test and Pearson Chi-Square test. *P* value ≤0.05 defined a significant difference between the compared variables. 

The study was approved by the Danish Data Protection Agency, file number 2010-41-5205.

## 3. Results

**Figure 1:**
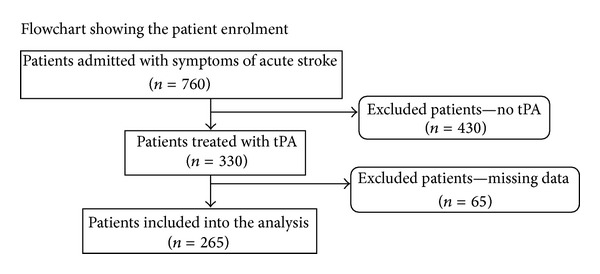
The registry was based on patients from a well-defined catchments area admitted with symptoms of acute stroke to Copenhagen University Hospital, Bispebjerg, from April 2009 to December 2011. Patients treated with tPA and no missing or undecipherable values of admission systolic blood pressure, admission-NIHSS, and prehospitalization mRS were included into the analysis; patients not treated with tPA or with missing or undecipherable values were excluded from the analysis.

Seven hundred and sixty patients were admitted and evaluated for thrombolysis treatment in the study period. Two hundred and sixty five (app. 35%) were treated with tPA and had decipherable systolic blood pressure values on charts (Figure 1). These patients were divided into 4 blood pressure quartiles and included into the analysis. No significant difference in age or gender was observed between the 4 groups (Table 1). Likewise no difference was observed between the included group (*n* = 265) and the excluded group (*n* = 65) concerning mean age, being 68 years (SD 13.7) versus 66 years (SD 13.8), respectively. Also no difference was found regarding NIHSS at admission, being 8.5 (SD 6.6) versus 8.6 (SD 5.9) and post-thrombolysis NIHSS, being 5.6 (SD 6.6) versus 4.5 (SD 5.4), respectively.

**Figure 2:**
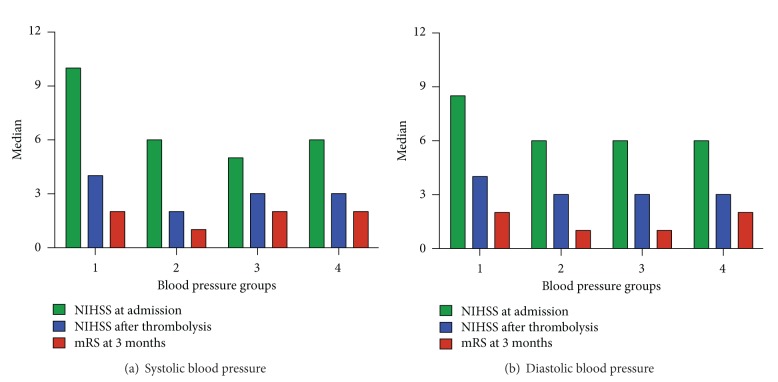
NIHSS at admission, NIHSS after tPA, and mRS after 3 months in the 4 systolic and diastolic blood pressure groups. A trend in poorer outcome is seen in patients with lowest and highest systolic and diastolic blood pressure. Outcome is represented with NIHSS at admission, after tPA and mRS after 3 months.

A trend towards a negative influenced short and long-term outcome was seen in both ends of spectrum concerning admission blood pressure. Low (group 1) as well as high (groups 3 and 4) admission blood pressure thus presented poorer outcome parameters compared with the group who showed admission values between 140 and 160 systolic (group 2) (Figure 2). This trend in outcome was repeated virtually identically in the comparison with diastolic blood pressure groups and both thus present U-shaped graphics (Figure 2).

Patients with low admission blood pressure (group 1) were alone in experiencing nonsignificant drop in systolic and diastolic blood pressure values during the first 12 hours after ictus (Table 2). This is in contrast to the patients with high admission blood pressure (groups 3 and 4) who experienced the largest drop in systolic and diastolic blood pressure values but still showed persisting elevated values after 12 hours (Table 2). 

**Figure 3:**
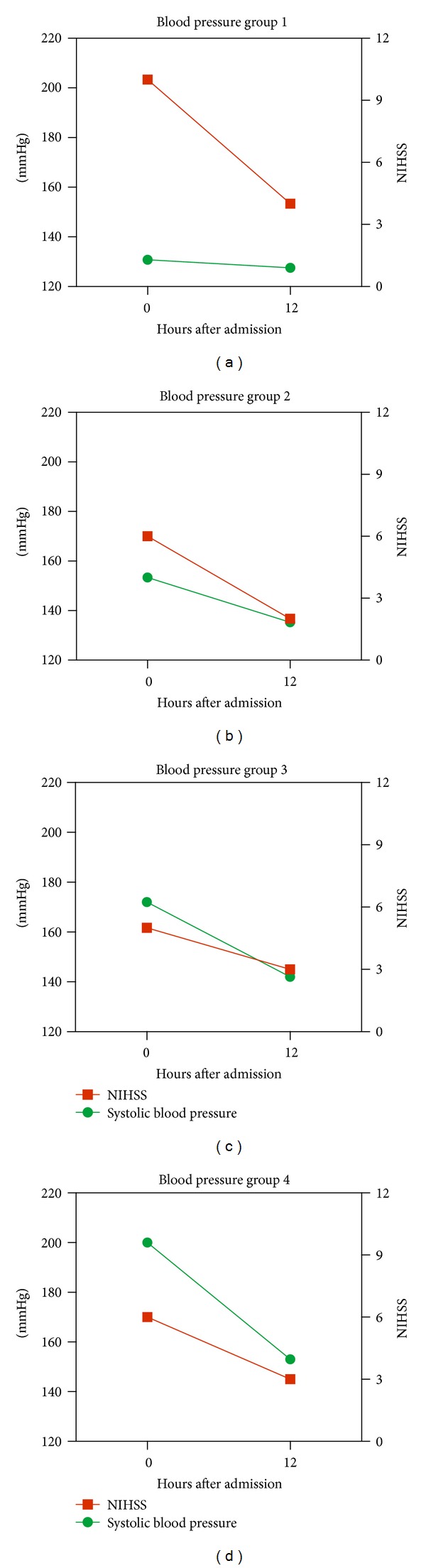
The association between NIHSS after tPA, representing short-term outcome, and the development in the systolic blood pressure groups within the first 12 hours after admission.

**Figure 4:**
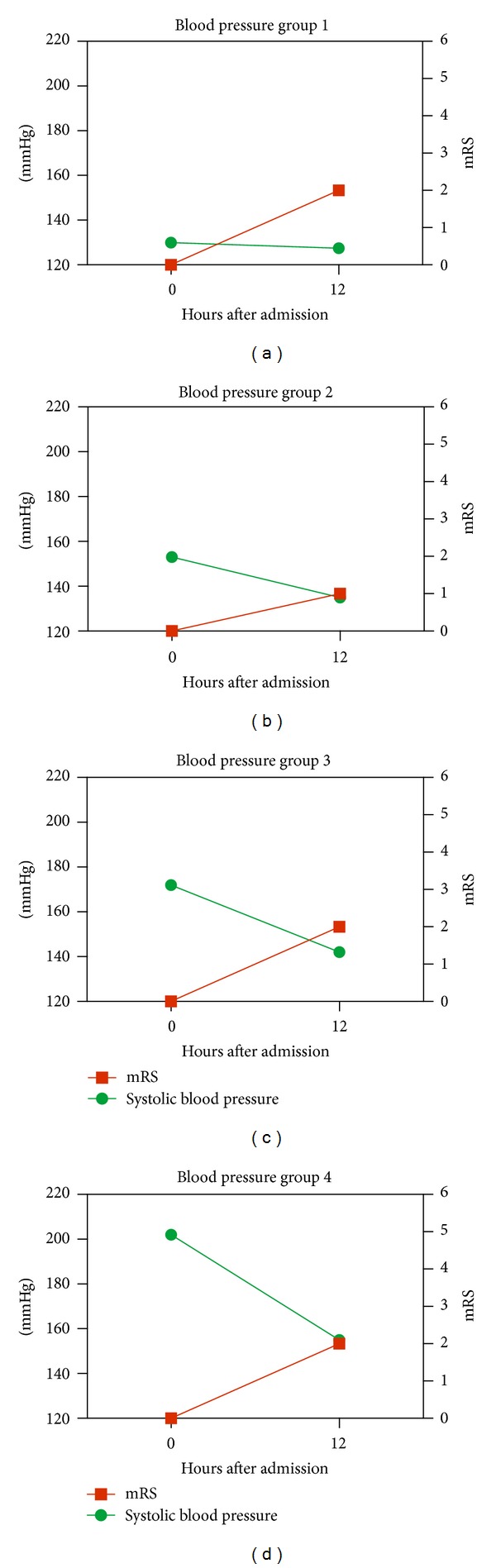
The association between mRS after 3 months representing long-term outcome, and the development in the systolic blood pressure groups within the first 12 hours after admission. The blood pressure group 2 has the lowest long-term outcome value (mRS = 1) compared to the other blood pressure groups.

Opposed to these, patients with admission blood pressure values between 140–160 systolic (group 2) experienced a moderate decrease in systolic and diastolic blood pressure values and showed levels closest to the normal spectrum after 12 hours. This tendency was associated with better short and long-term outcome (Figures 3 and 4).

Patients with mRS at 3 months of 0–2 experienced an average systolic decrease in admission blood pressure, which was 10 mm Hg higher than patients with mRS 3–6.

The presence of cardiac premorbidity in the group with low admission BT was significantly higher than in the other groups (Table 3). Preexistent hypertension was significantly higher in the 2 groups with the highest admission blood pressure, which was also the case for alcohol consumption (Table 3). No other significant differences in the presence of risk factors in the different groups were found.

## 4. Discussion

In the present study we demonstrated both a trend towards poorer short- and long-term outcome in groups with low as well as high admission blood pressure. A trend with a higher decrease in blood pressure after 12 hours in the quartile group with values interpretable as resulting from a physiological stress response was also found. 

Poor outcome in patients with low admission blood pressure has previously been identified as resulting from cardiac comorbidity inhibiting elevation of acute blood pressure levels [1, 2, 13, 14]. Similar poor outcome in patients with high admission blood pressure can be explained as a result of preexisting hypertensive disease with associated spread of complications [1, 2, 15–17]. Decrease in admission blood pressure in the hours after ictus from acutely elevated levels associated with physiological stress towards normalisation can be explained by the capacity of autoregulation in patients without significant premorbidity and mental stress in the acute phase of stroke [7]. These patients had an overall tendency towards better outcome corroborating previous findings [1, 13]. 

A number of factors influence the outcome after thrombolysis and among these blood pressure levels are only one among many. In accordance with this, we describe the findings in the present study as an association and not a stand-alone causal factor.

The possible presence of differences between factors, such as age, between the different groups with accentuated admission blood pressure, has been examined. No significant difference between the median age of the patients in the four blood pressure groups was observed and very broad confidence intervals were present. A slight tendency towards increasing age could be observed with increasing admission blood pressure, but this was not confirmed by statistical comparison between the groups.

The strength of this study is the homogeneous study population of stroke patients with symptoms within the first 4.5 hours. All patients were evaluated by the same neuro-radiological and neurological senior consultants and were monitored closely in an advanced stroke care unit. This study has limitations mostly due to missing values, which however can be contributed to random error why the study population remains representative. However, a comparison between the 65 excluded patients and the 265 included patients regarding median age, admission NIHSS, and postthrombolysis NIHSS showed no significant difference—or trends—between patients in the analysis and patients with missing values.

Results from the present study suggest what previously has been reported about the need for an extensive workup for stroke patients with a low admission blood pressure and suspected cardiac comorbidity [14]. Likewise patients with severe increased blood pressure extending after the acute phase should undergo examination for treatable underlying hypertension knowing that high blood pressure levels after stroke increases the risk of recurrent stroke [18]. Previous trial has found significant association between reduction in blood pressure and reduced stroke recurrence; however, the initiation of the blood pressure treatment was initiated 2 weeks after ictus [19]. 

Additional studies regarding this complicated area are needed like the presently ongoing trial, ENOS, aiming to investigate the effect of blood pressure decrease in hypertensive stroke patients within the first 7 days after symptoms debut [12].

## 5. Conclusion

Admission blood pressure co-variates with the short- and long-term outcome after stroke. Especially a low or high admission blood pressure can indicate a cardiac comorbidity or preexisting hypertension, where close monitoring and further examinations are requested to minimize poorer outcome. Interventional trials especially the ENOS trial will help clarifying if active blood pressure reduction affects patients' outcome significantly. 

## Figures and Tables

**Table 1:** The blood pressure groups divided into quartiles.

Systolic	*N* = 265	Range (mm Hg)	Mean age (SD)	Male gender (%)
BP group 1	69	≤143.0	65.5 (±15.1)	35 (50.7)
BP group 2	67	143.0–163.0	67.6 (±14.4)	41 (61.2)
BP group 3	63	163.0–181.5	69.3 (±11.6)	38 (60.3)
BP group 4	66	≥181.5	71.0 (±13.0)	41 (62.1)
*P* value			0.803	0.501

Diastolic	*N* = 259	Range (mm Hg)	Mean age (SD)	Male gender (%)

BP group 1	70	≤80.0	71.5 (±14.7)	33 (48.5)
BP group 2	64	80.0–90.0	65.2 (±15.1)	38 (59.4)
BP group 3	61	90.0–103.3	66.6 (±12.4)	41 (60.3)
BP group 4	64	≥103.3	69.9 (±11.7)	42 (65.6)
*P* value			0.388	0.238

No significant difference in age or gender was observed.

The missing values occurred due to an automatic archiving error involving follow-up blood pressure data from random cohort patients included through the study period making these undecipherable.

**Table 2:** Decrease in blood pressure from admission time to 12 hours after admission.

	Group 1			Group 2			Group 3			Group 4		
	Mean	*N*	SD	Mean	*N*	SD	Mean	*N*	SD	Mean	*N*	SD
At admission^1^	131	69	11.97	153	67	5.99	172	63	4.93	203	66	16.01
After 12 hours^1^	128	37	16.34	135	37	17.74	143	35	16.67	155	47	20.56
*P* value	0.2			<0.001			<0.001			<0.001		

At admission^2^	72	70	6.42	86	64	2.62	97	61	3.68	114	64	10.64
After 12 hours^2^	73	38	13.49	73	29	13.32	78	39	12.19	82	47	13.99
*P* value	0.5			<0.001			<0.001			<0.001		

^1^Systolic blood pressure.

^2^Diastolic blood pressure.

The blood pressure is significantly declined in quartile group 2 to 4, though still remaining above recommended levels in quartile groups 3 and 4. A nonsignificant decline is observed in quartile group 1.

**Table 3:** Previous medical history and risk factors in the blood pressure groups.

	Group 1		Group 2		Group 3		Group 4		*P* value
	*N*	%	*N*	%	*N*	%	*N*	%	
AFIB^1^	28	40.6	21	31.3	19	30.2	16	24.2	0.233
AFIB^2^	19	27.9	20	31.3	25	36.8	20	31.3	0.739
Previous MI^1^	6	8.7	4	6.0	8	12.7	4	6.1	0.470
Previous MI^2^	8	11.8	2	6.3	10	14.7	0	0.0	0.013
Hypertension^1^	34	49.3	37	56.1	45	71.4	54	83.1	<0.001
Hypertension^2^	41	60.3	33	52.4	46	67.6	49	77.8	0.021
Systolic									
No alcohol	18	27.3	15	24.2	11	19.6	16	26.7	0.891
<14/21 units	40	60.6	41	66.1	34	60.7	36	60.0	
>14/21 units	6	9.1	4	6.5	8	14.3	7	11.7	
Diastolic									
No alcohol	21	33.9	18	29.0	12	19.7	9	15.5	0.049
<14/21 units	35	56.5	39	62.9	35	57.4	41	70.7	
>14/21 units	4	6.5	4	6.5	9	14.8	8	13.8	

^1^Systolic blood pressure.

^2^Diastolic blood pressure.

AFIB and previously myocardial infarction was observed with highest prevalence in quartile group 1 and hypertension and alcohol consumption appeared with highest prevalence in quartile group 4.
